# The innovative technology of dough preparation for bread by the accelerated ion–ozone cavitation method

**DOI:** 10.1038/s41598-023-44820-1

**Published:** 2023-10-20

**Authors:** Auyelbek Iztayev, Talgat Kulazhanov, Galiya Iskakova, Mariam Alimardanova, Saule Zhienbaeva, Baurzhan Iztayev, Sholpan Tursunbayeva, Madina Yakiyayeva

**Affiliations:** Almaty Technological University, 100 Tole bi Str., 050012 Almaty, Kazakhstan

**Keywords:** Engineering, Mechanical engineering

## Abstract

Due to the fact that bakery, pasta and flour confectionery products are produced mainly from premium or first-grade flour, which is poor in the content of nutrients and fiber, the issue of developing technology for new types of flour products based on whole-ground flour of different fineness is very relevant and in demand. In the production of wholemeal flour, all parts of the whole grain are used—germ, grain shells, and endosperm. Also, recently the shortage of quality wheat has been growing. Therefore, the use of whole-milled flour from low-class wheat varieties will solve the problem of meeting the needs of the population. Using ion–ozone technology for preparing bread, high-quality bakery products from third-class flour with high nutritional and biological value were obtained. Using the obtained system of equations and constraints, the optimal modes of ion–ozone cavitation processing of dough were determined by a nonlinear programming method, which, subject to all the constraints (limitations) on the dough quality, provided the maximum dough strength of y_2_ = 181.0% and the dough parameter values of C × 10^–4^ = 25 units/mg, P = 1 atm, and τ = 5 min, which, in compliance with all constraints (restrictions) on the bread quality, provided a maximum volume of z_11_ = 232.1 cm^3^. A new innovative technology was created to increase productivity, efficiency and shorten the preparation time of bread. The method of making bread with the effect of ion–ozone cavitation of dough is very important for the bread industry, which affects the effectiveness of whole wheat flour obtained from the lower class of wheat, increases the quality of bread, shortens the technological processes of production, and increases labor productivity indicators. This method increases the economic efficiency of bread-making industries and the productivity of bread.

## Introduction

The main requirement of flour production is paying much attention to the flour and bread properties of wheat. Over the next decade, the production volume of high-quality wheat of the first and second classes will decrease by 8–10%, that of the medium or third class will comprise 70%, and that belonging to the fourth and fifth classes will increase by 20%^1,2^. Wheat classification provides for the main 5 classes. Classes from the first to the third are combined into group “A”, and the letter “B” denotes wheat of the fourth and fifth classes. Assignment to one group or another depends on the content of basic substances in the grains:

- protein—protein necessary for nutrition.
- gluten—improves baking properties.

1st class wheat contains 40% protein and 30% gluten, 2nd class wheat contains 13% protein and 27% gluten. The composition allows the use of grain in bakery production. Grade 3 wheat contains 12% protein and 23% gluten. 1–3 classes of wheat are included in group “A”. This is food wheat, which is used in the flour-grinding and baking industries.

4th grade wheat contains 18% protein and 11% gluten and 5th grade wheat contains 18% protein and 10% gluten. Classes 4 and 5 are included in group “B”. Usually these are hard varieties, which are also used for making cereals and pasta, but, unlike group “A”, they require saturation with strong varieties. The problem is that Group B varieties lack their own amount of gluten and proteins. These classes are also used for non-food purposes^3–7^.

Human consumption of high-quality wheat continues to increase, with the greatest growth expected in Asia, where the regional total accounts for almost 60% of global food use. Also, according to experts, the ongoing processes of global warming on the planet are affecting the decrease in the yield of many agricultural crops, in particular wheat. Therefore, it is necessary to use low-grade wheat for the production of bakery products^8–10^.

In baking technology, the process of proofing the dough is key, as it ensures the porosity, taste, and aroma of the finished product. In baking, biological, chemical, and mechanical methods are utilized for relaxing the dough depending on the variety. The peculiarity of the biological method of dough proofing is that sugars added to the dough are fermented to produce alcohol and gas. Carbon dioxide formed in the dough provides a porous structure^11–13^.

When utilizing the biological method, the dough must be relaxed for a sufficient amount of time to ensure the required quality of the finished product. During this method, several biochemical and microbiological processes take place in the dough, resulting in the accumulation of substances that confer porosity, volume, taste, and aroma on the final product. The biological method, despite its inherent technological shortcomings and economic costs, has been the main method of dough preparation for bakery products for a long time. Compressed, dried, active yeast, and fermented milk are used as biological leavening agents. In addition to compressed yeast, liquid yeast prepared in the bakery is utilized^14–17^.

The dough preparation method is chosen depending on the type and grade of flour being processed, its baking properties, the loosening method and the equipment used. Wheat flour dough is prepared with pressed, dry and liquid yeast, as well as with yeast milk obtained directly from the yeast factory. Obtaining high-quality flour from wheat of the third, fourth, and fifth classes is needed to make bread suitable for consumption. To solve this problem, a new technology of innovative bread making must be created, producing high-quality bread from different types of flour^18–20^. It is technologically possible to produce bread even from flours of slightly lower quality. Moreover, the time required to make bread through the use of traditional technology is up to 4–5 h, which reduces productivity, worsens the social situation, and does not ensure the baking of bread from low-quality flour. In this regard, the use of yeast, including a biological method, prolongs the time of the nanoproduct emergence and has a low probability of improving health^21–23^.

A considerable advantage of wholemeal bread is the amount of dietary fiber it contains. Dietary fiber promotes colon function and health, prevents constipation, and reduces the risk of colon cancer^24–26^. Yeasts contribute significantly to the aroma of bread by forming organic acids, alcohols, ketones, aldehydes, esters, and sulfur-containing compounds, including simple, non-dividing yeasts. Yeast-based bread products can be included in the diets of patients with gluten enteropathy in the case of a properly designed technological scheme and after testing whether the patient’s body can accept such products^27–29^.

Dough preparation methods can be multi-phase, including a pre-preparation stage and the preparation of special semi-finished products, which may differ in moisture content and microflora composition. Methods of preparing the dough can also be single-phase, including unpaired and accelerated methods. The essence of accelerated cooking methods is to strengthen the microbiological, colloidal, and biochemical processes occurring when cooking the dough. It is advisable to utilize quick methods in the production of bread and buns from wheat flour of high and first grade^30–32^.

Taking into account the need to solve these problems, new techniques of developing modern technologies must be proposed for the complex enrichment of the key nutrients of bread products, along with a new technology for baking bread with high nutritional and biological value based on using flour obtained from the lower classes of soft wheat with ion–ozone cavitation and ion–ozone water. To this end, research and development are required^33–35^.

Changes in socioeconomic conditions have led to considerable changes in the social status of various groups of the population. Non-observance of the main criterion of rational nutrition (balance) of the population is a major reason for the widespread of cardiovascular and oncological diseases, obesity, diabetes, allergies, and other diseases that determine demographic processes and public health indicators^36–39^.

In order to solve the scientific problem, a theoretical analysis of the principles of biological and mechanical kneading of the dough and the final products of the resulting processes was carried out, the features were identified, and a scientific basis was given for the use of mechanical kneading of semi-finished products. The physicochemical characteristics of foam masses, functional properties of the main components of flour and their role in the formation of stability of foaming and dispersion systems, and the rheological properties of dough are presented^40,41^.

There are known methods for mechanically loosening dough by churning part of it^42,43^, which consists of the following: part of the dough (in a relatively liquid and cold state) is churned for 5 min in a special churning machine of a durable and heavy design. After a short break, the kneaded mass is fed into a conventional kneading machine, in which the dough is kneaded, which is then sent for cutting and baking. However, these methods were used only for preparing dough from wheat flour and were not used in industry.

An analogue for the creation of innovative equipment and technology for functional bakery products with a shortened production cycle is the development of technology for aerated functional bread from wheat flour by scientists from the Voronezh State Technological University of Engineering Technologies^44^. The essence of the developed technology is as follows: in the first stage, the preparation of raw materials for production and dosing is carried out, in the second stage, the components are mixed for 9–10 min at a rotation speed of the kneading body of 5 s^−1^ in a HVA mixing machine, at the third stage, the dough is kneaded under pressure of 0.40 MPa for 10 min at a rotation speed of the kneading body of 10 s^−1^ in a hermetically sealed 2-chamber whipping installation made from first-grade wheat flour and additional raw materials according to the recipe; at the fourth stage, the kneaded dough is divided and sent for baking, The fifth stage involves packaging and storage.

In recent years, ozone, ions, ozone, ion–ozone and electronic technology, which have a number of advantages over special additives and technologies, are increasingly being used in the food industry. The use of ion–ozone technology agents with many useful properties (bactericidal, redox, etc.) in food production is the latest trend and represents a promising direction in food production. Currently, scientists of the Almaty Technological University are researching the use of ozonated, ionized and ion-ozonated water in the production of flour, bakery, pasta, flour confectionery products from wheat flour and flour from a mixture of wheat, grains, oilseeds and legumes, allowing to improve the quality, safety and environmental cleanliness of finished products.

Ozone has several undeniable advantages over other disinfectants. In particular, ozone is utilized for inactivating bacteria, bacterial spores, fungi, and viruses and is much more effective than formaldehyde, chlorine, ethylene oxide, etc^45,46^.

When using ozone in the food industry, it is worth considering the characteristics of the effect of ozone on the product itself, the species composition of suppressed microflora, temperature, humidity, and other parameters that may affect the effectiveness of ozone use.

Ozone has a high redox potential and high rates of reactions with organic molecules, therefore it is an antimicrobial agent. Ozone is a strong disinfectant gas that is used for canning, it has a powerful antimicrobial effect against viruses, bacteria, mold and fungus, protecting food from decay. Ozone technology is given a lot of attention because the treatment does not leave a residual amount of ozone in food products. In addition, this method extends the shelf life of food products, which is a major concern for food manufacturers, and the food industry needs safe and environmentally friendly technologies^47^.

The use of ozone as a disinfectant instead of traditional agents such as chlorine is justified by its significant oxidizing properties. It is about 50% stronger than chlorine and therefore has a wide range of antibacterial properties. The bactericidal effect of ozone has been confirmed on a large number of microorganisms, including gram-positive and gram-negative ones, as well as on bacterial spores. The main advantage of ozone treatment is most likely the absence of residual amounts in the processed products and objects. Unlike chemical methods (formaldehyde, ethyl alcohol), which leave residues that may or may have carcinogenic properties, affecting human health, ozone treatment does not contain chemical residues. In addition, ozone treatment is a promising replacement for the traditional fumigation (SO_2_) used. It can also be applied to all types of food, from fruits, vegetables, spices, meats and seafood to beverages^48^.

A similar phenomenon at low concentrations of ozone is characteristic of some mold types that appear on fruits. Therefore, it is necessary to use ozonators and other parameters allowing the concentration of ozone to be measured and adjusted over a wide range, directly in the ozone generator and in automatic mode, which supports the modes set by the operator. The initial effect of the optimum concentration of ozone on mold is the inhibition of its growth. The effect is immediate, particularly at the initial stage on the mold surface. Subsequently, processes of interaction with ozone result in the destruction of long-established cultures. Ozone primarily attacks accessible surface cells and penetrates slightly deeper^49–51^.

In the food industry, ozone is utilized for disinfecting premises, equipment, vehicles, containers, and packaging to enhance the sanitary and hygienic conditions of production. A low concentration of ozone is utilized in the bread production industry in order to enrich the nutrient medium, stimulate the growth of yeast fungi, and enhance the processes of making malt and yeast dough^52,53^.

During ion–ozone cavitation treatment (ozone concentration 2 g/m^3^ and molecular ions 1000 units/cm^3^ for 20 min, parameters may vary depending on humidity, grade, temperature, etc.) of flour and other components of plant raw materials, disinfection, clarification, a saturation of the product with oxygen and, based on quantum physical processes, the biological value of the product increases, ozonides are formed, which in further processes work as ozone, providing bactericidal and redox processes, gives increased acidity of the dough with a decrease in α-amylase and its inactivation temperature with a sharp decrease in enzymatic attackability of flour starch, which indicates the need to reduce yeast additives or eliminate them. The advantage of using an ion–ozone mixture allows, unlike other similar technologies, to process products without harmful impurities and radiation.

When processing the dough in the cavitator, an excess pressure of 2–6 ati is created (depending on the kneading parameters) and as a result of the pressure, cavitation bubbles are formed, filled with a hydro- ion–ozone mixture, while two phases are distinguished—expansion and collapse, which together form a complete thermodynamic cycle. In the pressure zone, hydrostatic pressure decreases to such an extent that the forces acting on the molecules of the liquid become greater than the forces of molecular bonds. As a result of a sharp change in hydrostatic equilibrium, the ion–ozonated liquid explodes, generating numerous tiny bubbles, while ozone, while still under pressure in the bubbles, has the ability to self-explode, in addition to a sharp drop in excess pressure in the cavitator from 2 atm and above to atmospheric pressure. As a result, the pores of the processed product increase.

Thus, the creation and implementation of a mechanical method of kneading the dough will allow the creation of new types of functional bread through the use of natural traditional and non-traditional raw materials ensuring the safety of bread products and a new technical and technological level of the industry, which will contribute significantly to the development of the country's economy.

The main stage of mechanical dough preparation is the accelerated production of a semi-finished product due to cavitation (excess pressure). The purpose of this study is to produce aerated dough from low-quality whole-grain flour, including third-grade flour, by mechanical loosening using an ion–ozone cavitation unit and baking bread.

## Research results

To determine the optimal option for preparing the dough, the responses of the dependence of physicochemical parameters on the factors of ion–ozone concentration, excess pressure and dough kneading time were studied.

A multifactorial experimental plan was devised through the use of the values of the ion–ozone (IO) water added during kneading, the amount of pressure during kneading, and the frequency of turning the dough according to the indicators of the upper, lower, and zero levels (Table 1).

**Table 1:** Mathematical regression equation describing the changes in indicators according to the plans of 2^3^ multifactorial.

No	Indicators	Encoded value	Factors and their values		
			x_1_- ion–ozone concentration, 10^4^ units/mg	x_2_- cavitation pressure during dough kneading, atm	x_3 -_ dough kneading time, min
1	Upper level	+	25	2	10
2	Zero level	0	15	1.5	7.5
3	Lower level	−	5	1	5
4	Interval		10	0.5	2.5

According to the research results, the values of the indicators presented in the upper, zero, and lower levels and the interval of multifactorial 2^3^ planned mathematical regression equations were selected. The following values are given in the table:

x1 is the ion–ozoneconcentration, 104 units/mg; x2 is the given cavitation pressure during dough kneading, atm; x3 is the dough-kneading time, min.

The following regression equations were obtained:

1 $${\text{y}}_{1} = {\text{f}}_{1} \left( {{\text{x}}_{1} ,{\text{ x}}_{2} ,{\text{ x}}_{3} } \right)$$

2 $${\text{y}}_{2} = {\text{f}}_{2} \left( {{\text{x}}_{1} ,{\text{ x}}_{2} ,{\text{ x}}_{3} } \right)$$

3 $${\text{y}}_{3} = {\text{f}}_{3} \left( {{\text{x}}_{1} ,{\text{x}}_{2} ,{\text{x}}_{3} } \right)$$

4 $${\text{y}}_{4} = {\text{f}}_{4} \left( {{\text{x}}_{1} ,{\text{ x}}_{2} ,{\text{ x}}_{3} } \right)$$

5 $${\text{y}}_{5} = {\text{f}}_{5} \left( {{\text{x}}_{1} ,{\text{ x}}_{2} ,{\text{ x}}_{3} } \right)$$

6 $${\text{y}}_{6} = {\text{f}}_{6} \left( {{\text{x}}_{1} ,{\text{ x}}_{2} ,{\text{ x}}_{3} } \right)$$

7 $${\text{y}}_{7} = {\text{f}}_{7} \left( {{\text{x}}_{1} ,{\text{ x}}_{2} ,{\text{ x}}_{3} } \right)$$

8 $${\text{y}}_{8} = {\text{f}}_{8} \left( {{\text{x}}_{1} ,{\text{ x}}_{2} ,{\text{ x}}_{3} } \right)$$

9 $${\text{y}}_{9} = {\text{f}}_{9} \left( {{\text{x}}_{1} ,{\text{ x}}_{2} ,{\text{ x}}_{3} } \right)$$

10 $${\text{y}}_{10} = {\text{f}}_{10} \left( {{\text{x}}_{1} ,{\text{ x}}_{2} ,{\text{ x}}_{3} } \right)$$

Optimal linear calculation models of physical and chemical indicators were created, and the amount of gluten y_9_ was taken as the objective function.

The objective function is as follows:

11 $${\text{y}}_{9} = {\text{f}}_{9} \left( {{\text{x}}_{1} ,{\text{ x}}_{2} ,{\text{ x}}_{3} } \right) \, \to \, \max$$

Here: max – maximum amount of gluten.

The limit function is as follows:

12 $$6.0 \, \le {\text{ y}}_{1} = {\text{f}}_{1} \left( {{\text{x}}_{1} ,{\text{ x}}_{2} ,{\text{ x}}_{3} } \right) \, \le \, 10$$

13 $$1.0 \, \le {\text{ y}}_{2} = {\text{f}}_{2} \left( {{\text{x}}_{1} ,{\text{ x}}_{2} ,{\text{ x}}_{3} } \right) \, \le \, 2.0$$

14 $$50.0 \, \le {\text{ y}}_{3} = {\text{f}}_{3} \left( {{\text{x}}_{1} ,{\text{ x}}_{2} ,{\text{ x}}_{3} } \right) \, \le \, 65$$

15 $$1.45 \, \le {\text{ y}}_{4} = {\text{f}}_{4} \left( {{\text{x}}_{1} ,{\text{ x}}_{2} ,{\text{ x}}_{3} } \right) \, \le \, 2.0$$

16 $$9.0 \, \le {\text{ y}}_{5} = {\text{f}}_{5} \left( {{\text{x}}_{1} ,{\text{ x}}_{2} ,{\text{ x}}_{3} } \right) \le \, 12.0$$

17 $$40.0 \le {\text{y}}_{6} = {\text{f}}_{6} \left( {{\text{x}}_{1} ,{\text{ x}}_{2} ,{\text{ x}}_{3} } \right) \le 50.0$$

18 $$18.0 \le {\text{y}}_{7} = {\text{f}}_{7} \left( {{\text{x}}_{1} ,{\text{ x}}_{2} ,{\text{ x}}_{3} } \right) \le 40.0$$

19 $$1.0 \le {\text{y}}_{8} = {\text{f}}_{8} \left( {{\text{x}}_{1} ,{\text{ x}}_{2} ,{\text{ x}}_{3} } \right) \le 33.0$$

20 $$23.0 \, \le {\text{ y}}_{9} = {\text{f}}_{9} \left( {{\text{x}}_{1} ,{\text{ x}}_{2} ,{\text{ x}}_{3} } \right) \, \le \, 30.0$$

21 $$60.0 \, \le {\text{ y}}_{10} = {\text{f}}_{10} \left( {{\text{x}}_{1} ,{\text{ x}}_{2} ,{\text{ x}}_{3} } \right) \, \le \, 90.0$$

The value *y* for dough and those of bread indicators are indicated by the following letters:

t_i_.—quality indicators of dough.

z_i_.—quality indicators of bread.

Next, the influence of factors on the rheological and physicochemical parameters of dough and bread was studied.

### Determination of the optimal values of the regime parameters of ion–ozone cavitation processing of dough from whole-ground flour of the third class of wheat for its rheological properties

A series of experimental studies were conducted to optimize the modes of ion–ozone cavitation processing of the dough, with the aim of increasing its quality indicators.

The concentration of ion–ozone (C, U/mg), overpressure (P, atm), and the duration of treatment (τ, min) were taken as regime factors for dough processing. The rheological parameters of the dough (t_i_) after treatment with ion–ozone cavitation treatment were investigated.

In order to decrease the number of experiments and increase the reliability of the results obtained, methods of planning multifactorial experiments were utilized. The plan of complete three-factor experiments of the FFE-2^3^ type with experimental conditions is given in Table 2. To reduce the influence of uncontrolled parameters on the experimental results, the experiments were randomized through the use of tables of random numbers.

**Table 2:** Planning matrix and results of experiments to study the effect of ion–ozone cavitation treatment of dough on its quality indicators.

N	Factors					Quality indicators								
	*C·*10^–4^, units/mg		*P*, atm	τ, minutes		*t*_1_	*t*_2_	*t*_3_	*t*_4_	*t*_5_	*t*_6_	*t*_7_	*t*_8_	*t*_9_
Control sample						61	123	134	13.8	13.6	8.55	16.92	23.28	29.82
1	25	2			10	86	75	134	18.0	13.2	8.38	16.78	22.75	30.83
2	5	2			10	67	83	109	16.5	12.6	8.43	16.83	22.98	31.08
3	25	1			10	75	127	100	25.0	12.6	8.57	16.83	23.18	30.70
4	5	1			10	78	147	105	16.7	13.6	8.45	16.95	23.45	30.42
5	25	2			5	69	94	80	11.2	13.9	8.23	16.73	23.10	30.82
6	5	2			5	67	93	87	11.0	13.6	8.43	16.58	22.77	31.03
7	25	1			5	63	195	153	16.9	14.0	7.72	16.92	23.28	30.43
8	5	1			5	88	86	100	11.5	12.7	6.80	16.68	22.78	31.22

where: C—concentration of ion–ozone, units·10^–4^/mg; P—pressure, atm; τ—duration, minutes; *t*_1_—dough extensibility index, mm; *t*_2_—dough strength, J; *t*_3_—dough elasticity index, mm; *t*_4_—dough formation time, minutes; *t*_5_—dough stability, minutes; *t*_6_—kneading time, minutes; *t*_7_—gluten, mg/kg; *t*_8_—amylase, %; *t*_9_—viscosity, %

The rheological parameters of the dough were determined using the "Structometer ST-2" device according to its methodology. It is intended to determine the rheological characteristics of food media. The operating principle of the device is based on measuring the mechanical load on the indenter nozzle when introducing it at a given speed into a prepared product sample. Necessary the indenter is mounted on a strain gauge beam, moved in the vertical direction by means of a ball screw according to a given program. The selection, setting of the mode and display of information is carried out using a personal computer connected to the data acquisition board of the device via a USB interface. When determining the rheological properties of the analyzed media, it can be set as the speed of movement of the indenter and the loading speed of the product. Measured characteristics: elastic and plastic deformation, work of elastic deformation, rigidity, hardness, tensile strength, elastic modulus, ultimate loading force, adhesive stress, ultimate shear stress, viscosity, relaxation time of mechanical stresses, depth of implementation, etc.

Gluten indicators are determined according to GOST 27,839–2013^54^. The essence of the method for determining the amount of gluten. Isolation of raw gluten from dough, kneaded from flour and water and held in water for hydration and the formation of intra- and intermolecular bonds in the substances that form gluten (mainly proteins—gliadin and glutenin), followed by washing with the working body of a mechanized device (mechanized method) or with your palms (manual method) using water, which removes water-soluble substances from the dough, as well as starch and bran. The resulting gluten is weighed and the percentage of raw gluten relative to the weight of the analyzed flour sample is calculated. In the manual method, before weighing, remove excess water by squeezing between your palms. The essence of the method for determining the quality of gluten. Determination of the amount of compressive strain of raw gluten, molded into a ball, under the influence of a specific load for a specified time interval.

α-amidase was determined according to GOST R 51228-98^55^. The alpha-amylase activity of a product is equal to 1 unit if the enzyme extracted from 1 g of product in a volume of 1 dm^3^, under certain conditions, causes in one second the hydrolytic breakdown of 1.024 × 10^–5^ units of beta-terminal dextrin substrate, calculated per unit of available substrate. Beta-terminal dextrin is the product of the complete breakdown of starch by beta-amylase. The enzyme cleaves the substrate—beta-terminal dextrin (hereinafter referred to as final dextrin). During the reaction, at certain time intervals, aliquots of the reaction mixture are added to the iodine solution. A decrease in color intensity with increasing reaction time characterizes enzyme activity.

By processing the experimental results according to the algorithms and programs of sequential regression analysis developed at the Odessa Technological Institute of Food Industry^56,57^ for each flour quality indicator considered, the corresponding regression equations were obtained and are presented in Table 3. As can be noticed from the statistical characteristics, the equations according to the Fisher criterion at a significance level of *p* = 0.05 adequately describe the dependence of the studied indicators of the dough quality on the modes of its processing—the concentration of ion–ozone C_io_, excess pressure P, and duration of processing τ.

**Table 3:** Regression equations in natural variables and statistical characteristics of the dependences of the rheological properties of the dough on the modes of its ion–ozone cavitation treatment.

Regression equations in natural variables	Standard deviation		Fisher's criterion	
	Experimental	Inadequacy	Estimated	Critical
*t*_1_ = 104.59 − 5.73*C* + 1.60*CP* + 0.384*C*τ − 2.43*P*τ mm	2.50	8.29	11.01	19.16
*t*_2_ = 11.84*C* + 12.47τ − 2.95*CP* − 0.829*C*τ J	5.60	24.41	19.00	19.25
*t*_3_ = 238.37 + 0.875*C* − 101.00*P* − 16.80τ + 12.00*P*τ mm	5.40	19.33	12.82	19.16
*t*_4_ = 5.00 + 0.361*C* + 1.07 − 0.148*CP* minutes	0.55	1.05	3.68	19.25
*t*_5_ = 13.27 min	0.46	0.58	1.60	19.35
*t*_6_ = 8.13 min	0.40	0.60	2.22	19.35
*t*_7_ = 16.79 mg/kg	0.23	0.12	3.51	4.74
*t*_8_ = 23.04%	0.55	0.26	4.44	4.74
*t*_9_ = 30.82%	0.54	0.29	3.41	4.74

where: C—concentration of ion–ozone, units·10^–4^/mg; P—pressure, atm; τ—duration, minutes; *t*_1_—dough extensibility index, mm; *t*_2_—dough strength, J; *t*_3_—dough elasticity index, mm; *t*_4_—dough formation time, minutes; *t*_5_—dough stability, minutes; *t*_6_—kneading time, minutes; *t*_7_—gluten, mg/kg; *t*_8_—amylase, %; *t*_9_—viscosity, %

An analysis of the obtained equations demonstrated that such indicators of the processed dough quality including the dough kneading duration, the gluten and amylase content, and the dough viscosity in the studied range of changes in the factors C, P, and τ did not depend on ion–ozone cavitation treatment.

As a result of processing, only the dough extensibility index, dough strength, dough elasticity index, and dough formation time were changed. Moreover, the dough extensibility index, dough strength, and dough elasticity index depended on all three factors C, P, and τ, while the dough formation time depended only on the factors C and P.

It is worth noting that the paired mutual influences of factors had a statistically significant effect on the following noted indicators of dough quality:

- the extensibility index of the dough C and P, C and τ, and P and τ;
- the strength of the dough C and P and C and τ;
- the elasticity index of the dough P and τ;
- the time to the formation of the dough C and P.

At the final stage of the research, optimization of the modes of ion–ozone cavitation processing of dough from whole wheat flour of the third class was performed.

The dough strength was chosen as the objective function as follows (22):

22 $$t_{2} = 11.84C + 12.47\tau {-}2.95CP{-}0.829C\tau \to \max$$

Let us perform a more detailed analysis of the influence of factors C, P, and τ on the dough strength during its ion–ozone cavitation treatment.

**Figure 1:**
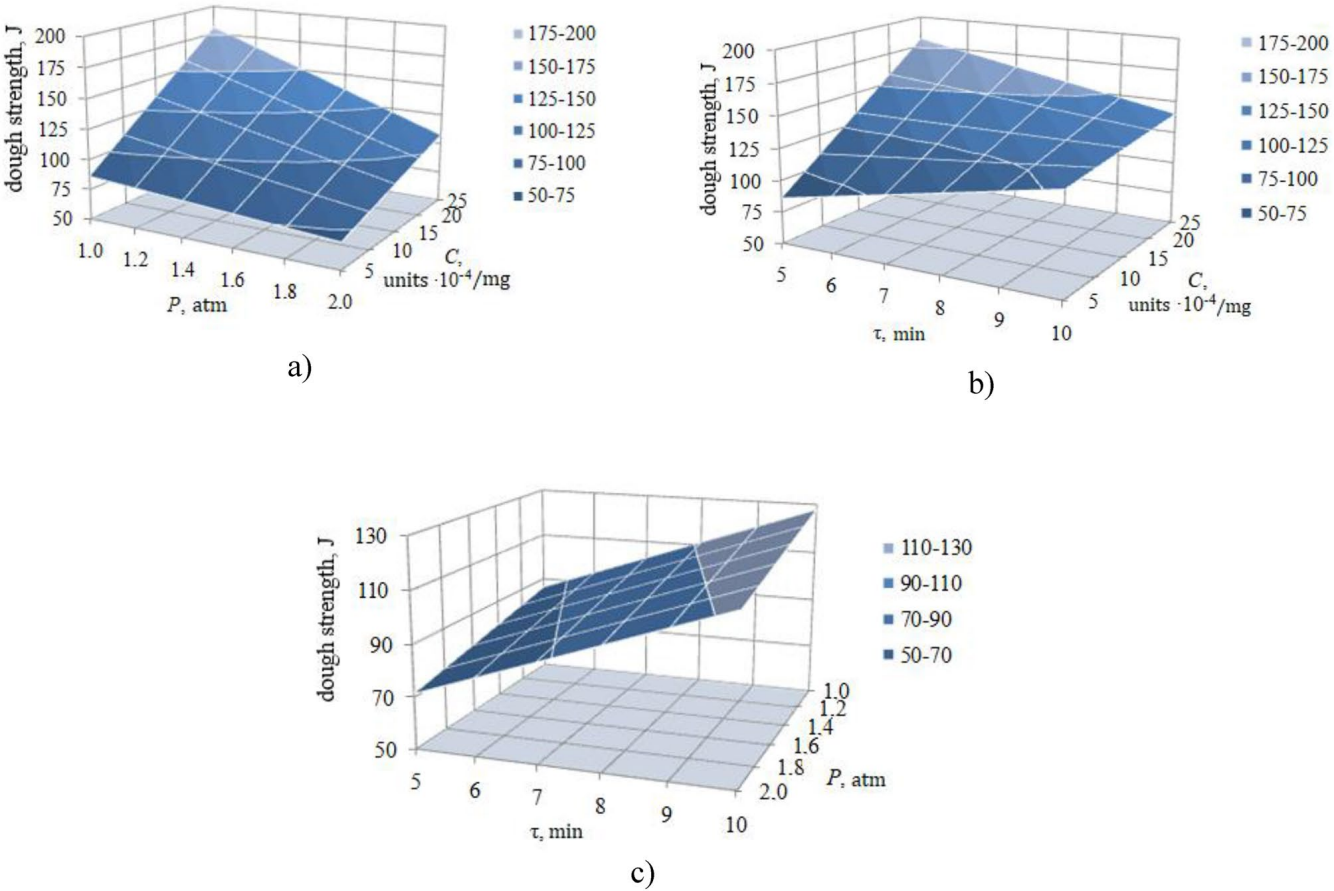
Response surfaces of dough strength dependence on factors C, P and τ: (**a**) τ = 5.0 min, (**b**) *P* = 1.0 atm, (**c**) *C*·10^–4^ = 25.0 units/mg.

As already noted, the strength of the dough depends on all three regime factors C, P and τ, and this dependence, due to the significance of the effects of pair interactions, is non-linear. Therefore, it is difficult to evaluate the influence of each factor separately on the strength of the dough, unambiguously and analytically using the regression equation, since the result of the influence of each specific factor on the strength of the dough depends on the values of the other factors. However, this can be performed simply and clearly by graphical interpretation of the regression equation—by the response surfaces built for each pair of factors and shown in Fig. 1.

Figure 1a depicts the impact of factors C and P on the dough strength during its processing τ = 5.0 min. Obviously, an increase in the C concentration, regardless of the pressure P, results in an increase in the dough strength, and an increase in pressure P, also regardless of the C concentration, reduces the dough strength. However, these changes in dough strength differ in magnitude. Thus, with an increase in the concentration of ion–ozone C, the dough strength increases in different ways—at P = 1.0 atm from 86.1 to 181.0% and at P = 2.0 atm from 71.4 to 107.3%. Pressure P also reduces the dough strength in different ways at different values ​​of C – at C = 5·10^4^ units/mg from 86.1 to 71.4% and C = 25·10^4^ units/mg from 181.0 to 107.3%, that is, almost 1.7×.

The impact of factors C and τ (at P = 1.0 atm) is more contradictory (Fig. 1b). It can be observed that at τ = 5 min an increase in C results in a significant increase in the dough strength from 86.1 to 181.0 J, and at τ = 10 min a change in C slightly changes the dough strength, only from 127.8 to 139.8 J. An increase in τ at different C changes not only the strength but also the direction of its influence. Thus, at C = 5·10^4^ units/mg, the dough strength increases from 86.1 to 127.8 J, but at C = 25·10^4^ units/mg, it decreases from 181.0 to 139.8 J.

The described complex character of the change in the dough strength is caused, as already noted, by the mutual influence of the factors C–P and C–τ. The absence of a significant coefficient of pair interaction between the factors P and τ results in a simple linear law of change in dough strength depending on pressure and dough processing time. This is clearly confirmed in Fig. 1c. An increase in both processing time τ and pressure P (at C = 25.0·10^–4^ units/mg) leads to a proportional increase in the dough strength. It can also be seen that the time factor changes the dough strength to a greater extent than the pressure factor—the processing time changes the dough strength by 1.46–1.53 times and excess pressure by 1.13–1.21 times.

For the rest of the rheological parameters of the dough, both dependent on the studied factors and independent, the following restrictions were chosen:

$$\begin{aligned} 50.0 \le t_{1} = & \, 104.59 \, {-} \, 5.73C + \, 1.60CP + \, 0.384C\tau {-} \, 2.43P\tau ,{\text{mm}} & \le 90.0 \\ 75.0 \le t_{2} = & \, 11.84C + \, 12.47\tau \, {-} \, 2.95CP{-} \, 0.829C\tau , \, J & \le 195.0 \\ 80.0 \le t_{3} = & \, 238.37 \, + \, 0.875C{-} \, 101.00P{-} \, 16.80\tau \, + \, 12.00P\tau ,{\text{ mm}} & \le 160.0 \\ 10.0 \le t_{4} = & \, 5.00 \, + \, 0.361C + \, 1.07 \, {-} \, 0.148CP,{\text{ minutes}} & \le 25.0 \\ 10.0 \le t_{5} = & \, 13.27,{\text{ minutes}} & \le 20.0 \\ 8.0 \le t_{6} = & \, 8.13,{\text{ minutes}} & \le 10.0 \\ 16.0 \le t_{7} = & \, 16.79,{\text{ mg}}/{\text{kg}} & \le 18.0 \\ 20.0 \le t_{8} = & \, 23.04, \, \% & \le 23.0 \\ 29.0 \le t_{9} = & \, 30.82, \, \% & \le 31.0 \\ \end{aligned}$$

Limitations on the range of changes in processing conditions were equal to the limitations on the range of changes in the modes of experiments:

5 units/mg ≤ *C*·10^–4^ ≤ 25 units/mg; 1 atm ≤ *P* ≤ 2 atm; 5 min ≤ τ ≤ 10 min

Using the obtained system of equations and constraints, the optimal modes of ion–ozone cavitation processing of dough were determined by the method of nonlinear programming:

*C*·10^–4^ = 5.0 units/mg, *P* = 1.0 atm, τ = 5.0 min,

which, subject to all constraints (limitations) on the dough quality, provide the maximum dough strength of *t*_2_ = 181.0%.

The obtained optimal values of all the studied rheological parameters of the dough are presented in Table 4.

**Table 4:** Values of rheological properties of dough under optimal modes of ion–ozone cavitation treatment.

Rheological indicators	min		opt		max
1. Dough extensibility index, mm	50.0	≤	37.28	≤	90.0
2. Dough strength, J	75.0	≤	181.0	≤	195.0
3. Dough elasticity index, mm	80.0	≤	135.2	≤	160.0
4. Dough formation time, minutes	10.0	≤	15.7	≤	25.0
5. Dough stability, minutes	10.0	≤	13.3	≤	20.0
6. Dough kneading, minutes	8.0	≤	8.1	≤	10.0
7. Gluten, mg/kg	16.0	≤	16.8	≤	18.0
8. Amylase, %	20.0	≤	23.0	≤	23.0
9. Viscosity, %	29.0	≤	30.8	≤	31.0

According to the results, the optimal values of the studied rheological indicators of the quality of the dough lie within the specified limits.

### Determination of the optimal values of the regime parameters of ion–ozone cavitation processing of dough from third-class whole-ground flour for its physical and chemical properties

To identify the optimal modes of ion–ozone cavitation processing of dough, which would make improving the bread quality possible, a series of experimental studies were conducted.

The concentrations of ozone ion (C, units/mg), overpressure (P, atm), and processing time (τ, min) were taken as regime factors for dough processing. The quality of bread after ion–ozone cavitation treatment was assessed by physicochemical quality indicators (z_i_).

In order to decrease the number of experiments and increase the reliability of the results obtained, the experiments utilized methods of planning multifactorial experiments. The plan of complete three-factor experiments of the FFE-2^3^ type with experimental conditions is shown in Table 5. In order to reduce the impact of uncontrolled parameters on the results of experiments, the experiments were randomized through the use of tables of random numbers.

**Table 5:** Planning matrix and results of experiments to study the effect of ion–ozone cavitation dough processing modes on bread quality indicators.

No.	Factors					Quality indicators										
	*C·*10^–4^, units/mg		*P*, atm	τ, minutes		*z*_1_	*z*_2_	*z*_3_	*z*_4_	*z*_5_	*z*_6_	*z*_7_	*z*_8_	*z*_9_	*z*_10_	*z*_11_
Control sample						3.9	0.45	37.7	2.2	3.46	51.1	5.3	65.0	46.0	4.6	231.9
1	25	2			10	4.20	0.47	34.8	2.03	3.19	46.5	4.6	59.5	44.0	4.2	209.5
2	5	2			10	4.37	0.48	33.0	1.94	3.04	50.3	4.8	60.7	42.0	4.5	183.7
3	25	1			10	3.89	0.5	37.8	2.21	3.47	47.7	5.2	62.7	43.0	4.3	218.7
4	5	1			10	4.68	0.52	34.9	2.04	3.2	49.5	3.8	58.6	41.0	4.3	225.3
5	25	2			5	4.52	0.48	33.7	2.01	3.33	53.0	5.1	62.9	45.0	4.2	216.6
6	5	2			5	4.64	1.09	30.6	1.78	2.8	46.9	4.6	63.3	43.0	4.1	205.6
7	25	1			5	4.59	0.5	35.4	2.03	3.24	52.5	5.8	57.9	44.0	4.6	227.5
8	5	1			5	4.49	0.52	32.0	1.87	2.94	54.5	5.5	59.1	42.0	4.4	229.6

where: C—concentration of ion–ozone, units·10^–4^/mg; P—pressure, atm; τ—duration, minutes; *z*_1_—protein, %; *z*_2_—fats, %; *z*_3_—carbohydrates, %; *z*_4_—ash content, %; *z*_5_—cellulose, %; *z*_6_—dough moisture, %; *z*_7_—dough acidity, degrees; *z*_8_—porosity, %; *z*_9_—bread moisture, %;* z*_10_—acidity of bread, degrees;* z*_11_—volume of bread, cm^3^.

The method for determining protein using the biuret reaction has good reproducibility and specificity, is relatively cheap and simple, and its use allows the study to be carried out both on analyzers (automatic and semi-automatic) and on a conventional photometer. The principle of the method is that proteins react in an alkaline medium with copper sulfate to form complex compounds colored violet. The intensity of the color, which is proportional to the amount of protein, determines its content in food products.

The mass fraction of fat in bakery products was determined by the extraction method with preliminary hydrolysis of the sample (arbitration method) according to GOST 5668^58^ and expressed as a percentage per 100 g of the whole product.

Carbohydrates: sucrose was determined by the permanganate method, starch by the polarimetric method (Evers)^59^. The fiber and cellulose contents were determined according to the Weende method using FIWE 3 devices^60^.

The ash content of bread was determined by the content of mineral salts (ash) in the flour, which remains after it is burned at high temperatures. The ash content of flour is expressed as a percentage and can vary from 0.3 to 2.5% depending on the type of flour and its production process. The higher the ash content of flour, the more fiber, vitamins and minerals it contains, but the quality of bread and other products decreases^61^.

The moisture content of the dough was determined according to GOST R 51414-99. The method consists of measuring and recording the consistency of the dough during its formation from flour and water, the development of the dough and the change in its consistency over time during the kneading process, using a valorigraph^62^.

The acidity of bread and dough was determined according to GOST 5670-96. The standard applies to baked goods, as well as low-moisture baked goods, and establishes methods for determining acidity. The degree of acidity is the volume in cubic centimeters of a solution of an exact molar concentration of 1 mol/dm^3^ of sodium hydroxide or potassium hydroxide required to neutralize the acids contained in 100 g of products^63^.

The porosity of bread was determined according to GOST 5669–96 using a Zhuravlev device^64^. Porosity is the ratio of the pore volume of the crumb to the total volume of the bread crumb, expressed as a percentage.

The moisture content of the bread was determined according to GOST 21094-2022. This standard applies to bakery products and establishes gravimetric methods for determining moisture (hereinafter referred to as the mass fraction of moisture) by drying a sample of the product in an oven and in an accelerated way—using a moisture analyzer (moisture meter). The method of drying a sample of a product in a drying cabinet is used when disagreements arise in assessing quality^65^.

The volume of bread was determined according to GOST 5667-2022. The volume of baked pan bread is determined using a P3-BIO brand meter. When determining the volume of bread using a P3-BIO brand meter, the filling container must be filled with prepared grain before starting work. At this time, the container should be in the upper position with the valve closed. Excess grain poured into the container is removed with a ruler. Then the grain is poured with the valve open from the filling container into the bread container, after which it is returned to the filling container again. Close the damper, return the container to its previous upper position and fill it with grain again. To determine the volume, place the bread container in the upper position and place the bread in it. The bread container is then lowered to the lower position. The grain moves from the top container to the bread container and fills it. The grain displaced by the volume of bread exits into a glass tube. After the grain stops settling in the tube, its level is counted on a scale, the readings of which correspond to the volume of the bread being measured^66^.

By processing the experimental results according to the algorithms and programs of sequential regression analysis for each flour quality indicator considered, the corresponding regression equations were obtained and are displayed in Table 6. As can be observed from the statistical characteristics, the equations according to the Fisher criterion at a significance level of *p* = 0.05 adequately describe the dependence of the studied indicators of the dough quality on the modes of its processing—the concentration of ion–ozone C_io_, excess pressure P, and duration of processing τ.

**Table 6:** Regression equations in natural variables and statistical characteristics of the dependences of the rheological properties of the dough on the modes of its ion–ozone cavitation treatment.

Regression equations in natural variables	Standard deviation		Fisher's criterion	
	Experimental	Inadequacy	Estimated	Critical
*z*_1_ = 4.676 − 0.00226*C*τ	0.071	0.173	5.93	19.33
*z*_2_ = 0.8404*P* − 0.02003*CPP* + 0.00303*C*τ − 0.05206*P*τ	0.0138	0.029	16.38	19.25
*z*_3_ = 31.625 + 0.140*C* − 2.000*PP* + 0.440τ	0.284	0.600	1.27	6.94
*z*_4_ = 1.989	0.095	0.128	1.83	19.35
*z*_5_ = 2.9169 + 0.01563*C*	0.095	0.147	2.41	19.33
*z*_6_ = 54.950 − 0.645τ	0.810	2.648	10.69	19.33
*z*_7_ = 5.900 − 0.130τ	0.170	0.555	10.67	19.33
*z*_8_ = 60.587	1.600	2.130	1.77	19.35
*z*_9_ = 43.00	1.600	1.309	1.49	4.74
*z*_10_ = 4.325	0.170	0.167	1.04	4.74
*z*_11_ = 246.700 − 15.627*P* − 0.324*CP* − 1.422*P*τ	3.300	7.195	4.75	19.25

Where: C—concentration of ion–ozone, units·10^–4^/mg; P—pressure, atm; τ—duration, minutes; *z*_1_—protein, %; *z*_2_—fats, %; *z*_3_—carbohydrates, %; *z*_4_—ash content, %; *z*_5_—cellulose, %; *z*_6_—dough moisture, %; *z*_7_—dough acidity, degrees; *z*_8_—porosity, %; *z*_9_—bread moisture, %;* z*_10_—acidity of bread, degrees;* z*_11_—volume of bread, cm^3^.

Obviously, all the regression equations obtained by the Fisher criterion adequately describe the dependence of the considered indicators of the processed dough quality on the conditions and methods of processing (factors C, P, and τ).

A brief analysis of the regression equations in Table 6 demonstrates that the concentration of ozone ion C affects only such physical and chemical indicators of bread quality including *z*_1_, *z*_2_, *z*_3_, *z*_5_, and *z*_11_. The pressure P (atm) influences the bread quality indicators *z*_2_, *z*_3_, and *z*_11_, while the dough processing time τ influences the quality indicators *z*_1_, *z*_2_, *z*_3_, *z*_6_, *z*_7_, and *z*_11_. Some indicators of bread quality (*z*_4_, *z*_8_, *z*_9_, *z*_10_) are not affected by the studied factors C, P, and τ at all.

Next, the problem of optimizing the technological modes of dough processing was formulated and solved, including the justification of the objective function, bilateral restrictions (limits of change) on the values of the factors, and the criterion for assessing the bread quality.

The bread volume z_11_ was chosen as the objective function of bread quality. In the problem, it is essential to identify the processing conditions (values of the factors C, P, and τ) that make achieving the maximum objective function possible, that is, the largest volume of bread z_11_.

Given the presence in many equations of significant coefficients of pair interactions (i.e., the nonlinearity of the objective function and quality assessment criteria), the search for optimal processing modes was performed through the use of nonlinear programming methods—the Newton method, which is part of the ‘Search for a solution’ procedure of the MS Office Excel package.

For a visual illustration of the nature of the dependence of the objective function on the factors influencing it, the corresponding response surfaces were constructed. When constructing these two-factor dependencies, the remaining significant factor included in the regression equation was fixed at the optimal level.

To optimize the bread volume (cm^3^), an objective function was adopted as follows:

23 $$z_{11} = 246.70{-}15.627P{-}0.324CP{-}1.422P\tau \to \max$$

Limitations for other quality indicators, both dependent on the studied factors and independent, were as follows:

$$\begin{aligned} 4.0\% \le z_{1} & = \, 4.676 \, {-} \, 0.00226C\tau , \, \% & \le 7.0\% \\ 0.45\% \le z_{2} & = \, 0.8404P{-} \, 0.02003CP + \, 0.00303C\tau \, {-} \, 0.05206P\tau , \, \% & \le 1.7\% \\ 29.0\% \le z_{3} & = \, 31.625 \, + \, 0.140C{-} \, 2.000P + \, 0.440\tau , \, \% & \le 37.3\% \\ 1.0\% \le z_{4} & = \, 1.989, \, \% & \le 2.0\% \\ 1.5\% \le z_{5} & = \, 2.9169 \, + \, 0.01563C,\% & \le 3.5\% \\ 47.0\% \le z_{6} & = \, 54.950 \, {-} \, 0.645\tau , \, \% & \le 52.0\% \\ 1.0{\text{ deg}} \le z_{7} & = \, 5.900 \, {-} \, 0.130\tau ,{\text{ deg}} & \le 5.3{\text{ deg}} \\ 50.0\% \le z_{8} & = \, 60.587, \, \% & \le 68.0\% \\ 41.0\% \le z_{9} & = \, 43.00, \, \% & \le 47.0\% \\ 4.0{\text{ deg}} \le z_{10} & = \, 4.325,{\text{ deg}} & \le 4.6{\text{ deg}} \\ 180{\text{ cm}}^{3} \le z_{11} & = \, 246.700 \, {-} \, 15.627P{-} \, 0.324CP{-} \, 1.422P\tau ,{\text{ cm}}^{3} & \le 250{\text{ cm}}^{3} \\ \end{aligned}$$

Restrictions on the range of changes in the conditions (modes) of dough processing were set as equal to the range of changes in the experimental conditions:

5 units/mg ≤ *C*·10^–4^ ≤ 25 units/mg; 1 atm ≤ *P* ≤ 2 atm; 5 min ≤ τ ≤ 10 min

Using the obtained system of equations, the optimal conditions (modes) of dough processing were determined by the nonlinear programming method:

*C*·10^–4^ = 25 units/mg, *P* = 1 atm, τ = 5 min,

which, subject to all the requirements (restrictions) on the bread quality, provide its maximum volume of *z*_11_ = 232.1 cm^3^.

The obtained optimal values of physical and chemical indicators of bread quality are shown in Table 7.

**Table 7:** Values of physical and chemical indicators of bread quality under optimal processing conditions.

Quality indicators	min		opt		max
1. Protein, %	4.0	≤	4.39	≤	7.0
2. Fats, %	0.45	≤	0.46	≤	1.7
3. Carbohydrates, %	29.0	≤	35.33	≤	37.3
4. Ash content, %	1.0	≤	1.99	≤	2.0
5. Cellulose, %	1.5	≤	3.31	≤	3.5
6. Dough moisture, %	47.0	≤	51.72	≤	52.0
7. Acidity of dough, degrees	1.0	≤	5.25	≤	5.3
8. Porosity, %	50.0	≤	60.59	≤	68.0
9. Moisture content of bread, %	41.0	≤	43.00	≤	47.0
10. Acidity of bread, deg	4.0	≤	4.33	≤	4.6
11. Volume of bread, cm^3^	180	≤	232.1	≤	250

**Figure 2:**
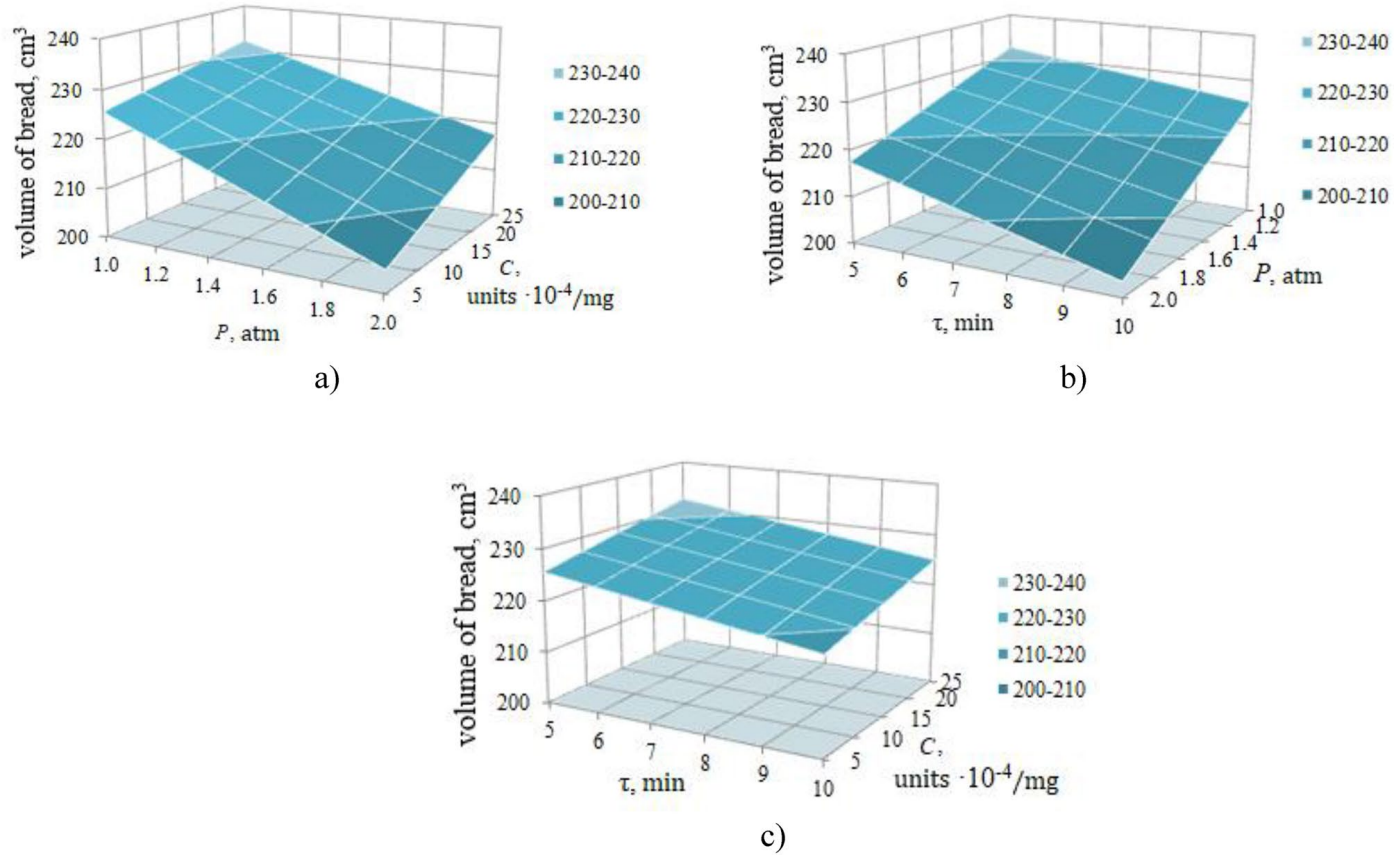
Response surfaces of bread volume dependence on factors C, P, τ: (**a**) τ = 5 min, (**b**) C = 25·10^4^, units/mg, (**c**) P = 1 atm.

For greater clarity of the dependence of the objective function on the studied processing conditions (factors C, P, and τ), the corresponding response surfaces of the influence of the studied factors were built and are displayed in Fig. 2.

Analyzing the response surface of the dependence of the bread volume on the factors C and P at τ = 5 min, Fig. 2a indicates that as the concentration of ozone ion C increases, the bread volume also increases. Moreover, the greater the pressure is, the more the volume increases, which is explained by the mutual influence of the factors C and P. Thus, at P = 1 atm, the bread volume with an increase in the concentration of ozone ion C from 5·10^4^ to 25·10^4^ units/mg increases by 6.46 cm^3^, and at P = 1 atm by 12.96 cm^3^.

If the effect of pressure on the bread volume is considered, then its effect is opposite to that considered—with an increase in pressure, the bread volume decreases, and at a lower concentration of ozone ion C, the decrease is more pronounced (21.12 cm^3^ vs. 14.64 cm^3^), emphasizing the greater influence of the force of pressure.

Figure 2b also clearly illustrates the mutual influence of factors τ and P on the volume of bread at C = 25·10^4^ units/mg. At τ = 5 min and with a reduction in P from 2 to 1 atm, the volume of bread increases by 14.64 cm^3^, and at = 10 min by 20.75 cm^3^. Similarly, the duration of dough processing τ manifests itself—with its reduction from 10 up to 2 min and P = 2 atm, the volume of bread increases by 14.22 cm^3^, and at P = 2 atm by 7.11 cm^3^.

As for the influence of factors C and τ on the volume of bread at P = 1 atm (Fig. 2c), due to the absence of their mutual influence, the change in the volume of bread occurs proportionally—as C increases, the volume of bread increases by 6.48 cm^3^, regardless of the duration of processing τ, and as τ increases, the volume of bread decreases by 14.64 cm^3^, regardless of the value of C.

Thus, the analysis of the influence of the considered factors C, P, and τ, complicated by the pairwise mutual influence of C–P and P–τ, confirmed the results of the optimization of the modes of ion–ozone cavitation processing of the dough, allowing the maximum volume of bread—232.1 cm^3^ to be reached.

## Discussion

Based on the ion–ozone cavitation effect of dough preparation, contributing to the improvement of the product quality, environmental conditions, and shortening of technological processes, the technology of production of bread products without yeast from wheat flour of three classes of ground from whole grains has been developed.

When the dough components are pressurized with compressed air, the semi-finished product is intensively saturated with air cells, the structural and mechanical properties of the dough are improved, and its volume and mass are reduced. This contributes to a reduction in the number of SH groups and the formation of S–S bonds in the protein structure, strengthening the protein structure.

Under the influence of proteolytic enzymes, the complex structure of the protein molecule is simplified, its ability to swell decreases, and the solubility of proteins increases. The main reaction catalyzed by proteolytic enzymes is also that they hydrolyze a part of auxiliary proteins closely related to the surface of starch granules.

The main reaction catalyzed by proteolytic enzymes is the hydrolysis of peptide bonds in proteins and peptide molecules. Polypeptides, peptides, and amino acids are formed as a result of protein hydrolysis under the influence of protease.

Protein molecules have reactive SH groups, which are able to oxidize under the influence of oxygen, when the dough components are pressed under pressure, the semi-finished product intensively saturates the air with oxygen. In addition, the action of enzymes (a-amylase and protease) on starch and flour protein contributes to the intensive formation of substances that determine the taste and aroma of bread. During this period, a number of products of enzymatic hydrolysis of proteins and starch (low-molecular nitrogenous substances, polypeptides, peptides, amino acids, and carbonyl compounds) participate in the formation of the taste and aroma of unfermented products, as well as participate in the reaction of melanoidin formation, which occurs during baking of white bread. As a result, melanoidins are formed.

Doughs were produced in an ion–ozone cavitation unit to reduce the duration and eliminate some stages of the technological process of dough preparation during the baking of bread and bakery products, as well as to increase the biological value of the processed products. Structured water was charged with ozone, atomic or molecular ions, and their mixtures and ozonized according to the technological process with cavitation. This process helps to lower production costs, ensure high processing speed, and guarantee product quality, environmental and biological purity, minimal energy consumption, reliability, and ease of use. When enhancing the process of baking bread and other products, it is important to obtain an environmentally friendly food product with improved quality through a simple and short technological process^67–69^.

The number of dough deformation cycles according to the frequency of rotation of the kneading body directly influences the dough quality. A link was established affecting the completeness of the process of forming the dough microstructure, its properties, and the bread quality^70–73^.

The ion–ozone cavitation technology of dough preparation allows us to completely avoid the process of fermentation and filling of dough (and therefore the appropriate equipment: dough kneading machine, dejas [kneading bread dough], fermentation vessels, and filling cabinet) to eliminate the use of pressed yeast when kneading dough. Thus, the use of ion–ozone cavitation technology in dough preparation allows a reduction in the duration of the technological process of bread production from 3 to 6 h, increasing labor productivity by 2–3×, increasing bread production by 14–18%, and enhancing the quality indicators of the finished product^74–77^.

The bakery industry is designed to provide the population with bread products in sufficient quantity and range to form a healthy and balanced diet. A review of patent and literary technologies for covering short-cycle bread for more than 10 years was carried out, and the methods of our research work were determined by rotating the dough intensively with high revolutions under air pressure, suitable for scientific and practical production, and further improving it. An analysis of the current state and development trends of the range of functional bakery products was conducted^78,79^.

The dough was taken by a new unit of kneading with innovative ion–ozone cavitation technology, and its optimal modes were created by mathematical processing.

Based on the ion–ozone cavitation technology for the dough preparation, which allows the removal of yeast according to the recipe, shortens the duration of the production process, enhances the quality of the finished product, increases labor productivity, and improves the socioeconomic indicators of baking enterprises, a new direction of production of bread products without yeast was created.

The use of ion–ozone water with numerous useful properties and ion–ozone cavitation technology of dough preparation in the production of bread products contributes to the creation of environmentally safe products.

By performing multifactorial planned experiments of each investigated physicochemical, biological, rheological, microbiological, and nutritional indicator with control modes of ion–ozone cavitation technologies, mathematical regression equations determining their changes were applied, and their statistical characteristics were revealed.

Scientific and technical documents of new innovative bread production technology were created, production was approved, and economic, social, and environmental efficiency were presented^80–82^.

In determining the possible technological parameters of dough kneading with innovative ion–ozone cavitation technology, a linear orientation model was created on the basis of regression equations, and all indicators of dough and bread were taken into consideration through the use of target and limit functions.

On this basis, production recipes, new assortments of unleavened bread products, and their technological modes were proposed.

Relative physicochemical, biological, flour, and bread properties were investigated, storage capacity and safety indicators were determined, and the scientific-practical basis for coating unleavened bread with the effect of cavitation was established.

For the first time, in order to increase the technological potential of low-class whole wheat flour, its amino acids and vitamin indicators were fully studied, and the possibility of obtaining high-quality bread with ion–ozone cavitation technology was revealed. To identify the possible ion–ozone cavitation winter cycle kneading technology regimes from whole wheat flour, linear orientation models were created based on mathematical regression equations, and the regimes of high physical, chemical, biological, and rheological properties, low microbiological indicators, stable safety, and a high-quality yeast-free bread coating were fully determined and proposed.

The intensification of the process is due to the fact that during the preparation of the dough, there is no need to use a pre-proofer and other machines used in traditional bread baking, which in turn allows a reduction in production areas, as well as reduces the time spent on making bread.

The use of an ion–ozone cavitation plant for accelerated dough preparation will improve the structural-mechanical, rheological and technological properties of aerated dough and products from it, reduce energy consumption and increase production productivity by reducing the duration of mixing and churning, automated dough division process^83,84^.

Ion–ozone cavitation treatment of dough allows you to shorten the production process, in particular, reduce the fermentation process of dough when preparing bakery products, and reduce the amount of pressed yeast when making dough. The use of cavitation treatment of dough makes it possible to reduce the duration of the bread production process from 3 to 6 h, significantly increase labor productivity by 2–3×, and increase bread yield by 14–18%.

The production of new types of bakery products based on environmentally friendly technologies using whole-milled flour and cavitation processing of dough will solve the following market problems:

- changing the structure of consumption of bakery products towards an increase in more functionally healthy products. This change in the consumption structure brings two positive results: the prevention of various diseases in the population and the involvement of additional raw materials in food circulation;
- short shelf life of flour products, which increases the number of returns and does not allow expanding the geography of product supplies.

## Materials and methods

### Raw materials

The following raw materials were utilized for preparing experimental samples: soft wheat flour of the third grade^85^ (was purchased from the Kazakh Research Institute of Agriculture and Crop Production on the basis of an agreement No. 20-04-13/C), table salt (GOST R 51574-2000)^86^, and drinking water (SanPiN 2.1.4.1074-01^87^).

### Methods of detection

Physicochemical and rheological indicators of dough and bread products were studied. Research on the determination of physicochemical and rheological indicators of dough, and bakery products was conducted at the Research Institutes of Food Safety and Food Technologies of Almaty Technological University. The moisture content was determined by the accelerated method according to GOST 9404-88. This International Standard applies to flours and bran and specifies the air-thermal method for the determination of moisture content. The essence of the method lies in the dehydration of flour and bran in an air-heating cabinet at fixed temperature and drying time parameters^88^. The composition of raw gluten was controlled following GOST 27839-88. This standard applies to wheat flour and establishes methods for determining the amount of gluten by washing it out of the dough using mechanical means or manually and the quality of gluten by measuring its elastic properties. Gluten is a complex of protein substances capable of forming a cohesive elastic mass when swollen in water. The results of measurements of the elastic properties of gluten are expressed in conventional units of the IDK-1M device and, depending on their value, gluten is assigned to the appropriate quality group^54^. α-amidase was determined according to GOST R 51228-98^55^. This standard specifies a colorimetric method for the determination of alpha-amylase activity in grains and cereal products ranging from very low to very high values. The method is also used to evaluate the activity of alpha-amylase in additives of fungal or bacterial origin. The protein mass fraction was determined according to GOST 10846-91. This standard applies to grain and products of its processing and establishes a method for the determination of protein. The essence of the method lies in the mineralization of organic matter with sulfuric acid in the presence of a catalyst with the formation of ammonium sulfate, the destruction of ammonium sulfate with alkali with the release of ammonia, the stripping of ammonia with water vapor into a solution of sulfuric or boric acid, followed by titration^89^. The fat content was determined according to GOST 29033-91. This standard applies to grain and products of its processing and establishes a method for determining fat. The essence of the method lies in the extraction of crude fat from the product with a solvent, the subsequent removal of the solvent, drying and weighing of the extracted fat^90^. The fiber mass fraction was determined according to the Wend method with the FIWE 3 analyzer. Also, other standard methods and techniques were used to study dough and bakery products.

### Devices and installations

Flour made from whole wheat grains was obtained by disintegration-wave milling in a disintegrator.

Before sending various standard or low-class wheat for grinding, the process of moistening the wheat in a certain amount was conducted so that the quality indicators of the flour obtained from the wheat were good. Moistening grain before grinding at flour mills is one of the most important conditions for improving the quality of products. With proper moistening, the grinding qualities of grain are improved, the load on flour-grinding machines is reduced, the ash content of flour is reduced, and as a result, the yield of premium flour increases.

According to the weight of wheat, the amount of water required for hydration is calculated by the following formula (24):

24 $${\text{V}}_{{{\text{water}}}} = \left( {{\text{W}}_{1} {-}{\text{ W}}_{2} } \right) \cdot {\text{V}}_{{{\text{flour}}}} /100$$

where W_1_ is the required humidity, %; W_2_ is the moisture content of different types of wheat during the same period of time, %; V_flour_ is the required amount of wheat of different varieties for grinding, kg.

**Figure 3:**
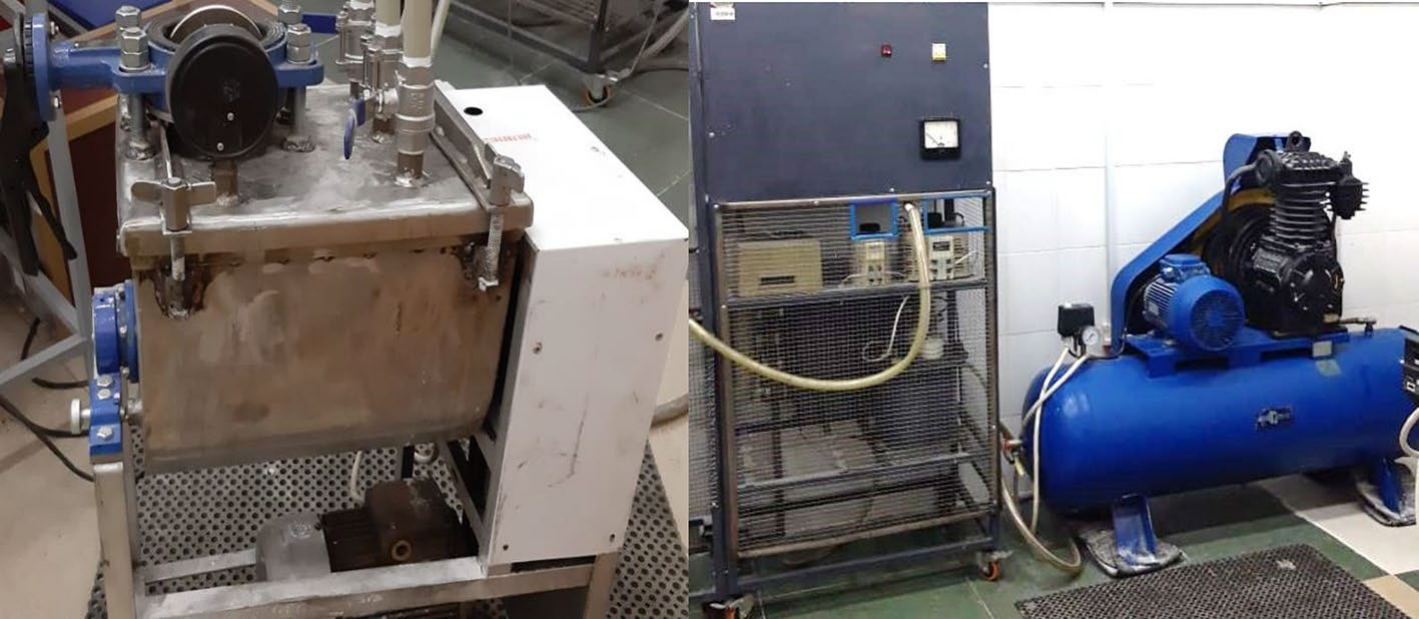
Laboratory ion–ozone cavitation unit for accelerated dough preparation.

To reduce the time and eliminate some stages of the technological process of dough preparation for baking bread and bakery products and to increase the biological value of processed products, they were produced in an ion–ozone cavitation unit (Fig. 3) using ion-ozonated water, saturated with ozone, atomic or molecular ions, and their mixtures, in accordance with the technological process – cavitation. The ion–ozone cavitation unit for dough preparation consists of units and mechanisms of control, regulation, and protection, integrated into a single unit by a common ion–ozone cavitator, ensuring the safety of the operating personnel. All knots and details of working bodies are made of food stainless steel. The electric motor has a frequency controller for the speed of rotation of the shaft. Laboratory ion–ozone cavitation plant for dough preparation has a capacity of 50 kg/h, dimensions of 1000 × 800 × 800 mm, and a plant weight of 50 kg. Standard materials, profiles, and electrical devices were utilized in the design of the laboratory ion–ozone cavitation unit for dough preparation.

Ion–ozone cavitation plant for accelerated dough preparation consists of a dough preparation tank, bubbling shaft, flour dispenser, water dispenser, ozone supply pipe, negative or positive air oxygen ions, ion–ozone mixture, overpressure sensor, electric wire, electric drive, fuses, frequency converter, cavitator lever.

The technological procedure of processing the receipt of the dough is as follows. The dough was obtained by mechanical pressure loosening in an experimental ion–ozone cavitation device for the preparation of dough with an accelerated cycle. The prescription dough components are fed through the feed opening into the kneading body of the batch mixer, in which the kneading body is installed, and driven by an electric motor using a speed variator. At the end of the loading, the kneading body of the kneading machine is hermetically sealed with a lid and the dough is kneaded for 5 min at a kneading body rotation frequency of 25 Hz. Then, ion–ozone cavitation air is supplied to the kneading chamber at a pressure of 2 atm and the dough is knocked down for 3 min at a rotation frequency of the kneading body of 25 Hz. When knocking down prescription components, the dough mass is saturated with air. The dough prepared in this way is a foamy mass with stable physical and chemical characteristics. The dough prepared in this way is a foamy mass with stable physicochemical characteristics. The duration of baking at a baking chamber temperature of 220 °C was 25–30 min, depending on the mass of the product.

Whipped yeast dough was prepared for a laboratory ion–ozone cavitation unit at 55%–56% humidity according to the recipes and regimes shown in Table 8.

**Table 8:** The recipe for whipped yeast bread made from third-class flour and the modes of cooking the dough.

No	Raw material and process indicators	Raw material consumption and process parameters
1	Wheat flour, third class, kg	10.0
2	Yeast for dough, kg	0.25
3	Table salt, kg	0.15
4	Vegetable oil, kg	0.02
5	Water, l	By calculation
6	Dough moisture, %	55–56
7	Duration of mixing, min	5
8	Output duration, min	3
9	Cavitation (excess pressure), atm	2
10	The frequency of rotation of the kneading body, Hz	25

## Statistical analysis

The data obtained during the experiments were processed using the mathematical method of variation statistics using the Statistika 10.0 developer: StatSoft, USA. Also, the data were analyzed using MS Excel for Windows version 10 Pro, 2010. The data collected during the study were subjected to independent testing, and questionnaires were conducted to assess the organoleptic characteristics of control and dough samples. The analysis process used absolute and relative statistical indicators and tabular and graphical methods for presenting the results. Values were estimated using mean and standard deviations. The experiment was conducted with three repetitions. Taking into consideration the presence of significant coefficients of pair interaction (i.e., the nonlinearity of the objective function and quality assessment criteria) in numerous equations, the search for optimal processing modes was performed through the use of nonlinear programming methods—the Newton method, which is part of the ‘Find a solution’ procedure in the MS Office Excel package.

## Conclusion

The work involved the optimization of ion–ozone cavitation treatment modes for dough made from whole-ground wheat flour of the 3rd class. Analysis of the obtained equations showed that the dough extensibility index, dough strength and dough elasticity index depend on all three factors C, P and τ, and the dough formation time depends only on factors C and P. Regression equations were obtained using the Fisher criterion, which adequately describes the dependence physicochemical and rheological indicators of the quality of processed dough and bread depending on the conditions and processing methods (factors C, P and τ). The results of the bread study showed that the concentration of ozone C ion affects only such physical and chemical indicators of bread quality as: z_1_—protein, %; z_2_—fats, %; z_3_—carbohydrates, %; z_5_—cellulose, % and z_11_—the volume of bread, cm^3^. Pressure P (ati) affects bread quality indicators z_2_—fats, %; z_3_—carbohydrates, % and z_11_—the volume of bread, cm^3^, and the duration of dough processing τ—for quality indicators z1—protein, %; z_2_—fats, %; z_3_—carbohydrates, %; z_6_—dough moisture, %; z_7_—dough acidity, degrees and z_11_—the volume of bread, cm^3^. Some indicators of bread quality (z_4_—ash content, %; z_8_—porosity, %; z_9_—bread moisture, % and z_10_—the acidity of bread, degrees) are not affected by the studied factors C, P and τ at all. Using an equation system and the nonlinear programming method, the optimal conditions (modes) for dough processing were determined, which, subject to all requirements (limitations) for bread quality, provide its maximum volume z_11_ = 232.1 cm^3^: C 10^–4^ = 25 units/ mg, P = 1 atm, τ = 5 min. Analysis of the influence of the considered factors C, P and τ, complicated by the pairwise mutual influence of C–P and P–τ, confirmed the results of the optimization of the ion–ozone cavitation treatment of dough, which allows achieving the maximum volume of bread.

## Data Availability

The datasets used and/or analyzed during the current study are available from the corresponding author upon reasonable request.
